# Virtual spaced-learning method, during COVID-19 for Pharm D students

**DOI:** 10.1186/s12909-023-04595-5

**Published:** 2023-08-24

**Authors:** Meysam Sharifdini, Mehdi Evazalipour, Zahra Hesari

**Affiliations:** 1 Department of Medical Parasitology and Mycology, School of Medicine, Guilan University of Medical Sciences, Rasht, Iran; 2 Department of Pharmaceutical Biotechnology, School of Pharmacy, Guilan University of Medical Sciences, Rasht, Iran; 3 Medical Education Research Center, Education Development Center, Guilan University of Medical Sciences, Rasht, Iran; 4 Department of Pharmaceutics, School of Pharmacy, Guilan University of Medical Sciences, Rasht, Iran

**Keywords:** Spaced learning, Mass learning, Virtual learning, Pharm D students, COVID-19 pandemic

## Abstract

**Background:**

The coronavirus (COVID-19) outbreak basically changed teaching methods across the world, and learning was almost replaced by virtual learning during the pandemic. Also, the spacing effect is one of the most well-established phenomena in the science of learning. Using temporal intervals for re-exposing learners to information over time (spaced learning) leads to more effective retention of knowledge compared to having information presented at a single time (massed learning). Hence, we designed a virtual spaced learning method to reap the benefits of virtual learning and spaced learning concomitantly.

**Methods/approach:**

An interventional semi- experimental survey among 66 Pharm D students was designed and implemented. Students were divided into two groups (spaced vs mass learning) in the national integrated virtual education platform (NAVID) as the matrix for teaching as well as evaluation. Classes were conducted in the following sequence: 1- answering the pre-test, 2- watching and listening to the educational content (separately for each group), 3- answering the post-test (*n* = 1). The pre/post-test consisted of 10 four-choice questions based on the Kirkpatrick Model extracted from the educational content.

**Results/outcomes:**

Findings revealed that the average score was not significantly different between the post-tests of the spaced learning and mass learning (7.26 ± 2.26 vs 6.5 ± 2.5) methods utilizing the independent t- test (*p* ≥ 0.05).

**Conclusions:**

Since no statistically significant improvement was observed in the virtual spaced learning group compared to the control group, it seems that clarifying the significant influence of the spaced learning strategy in pharmacy education requires longer period of study, or study on less complex or skill-based topics for further evaluation.

**Supplementary Information:**

The online version contains supplementary material available at 10.1186/s12909-023-04595-5.

## Introduction/background

The new coronavirus (COVID-19) in 2019 has basically changed the way teaching is practiced across the world [1]. Social distancing and limitations on gathering in order to minimize the spread of COVID-19 based on WHO recommendations, have seriously affected Pharm D student training [2]. Since the start of the pandemic, Pharm D education has been fundamentally renovated and almost all academic programs including lecturers and different assessments have turned to virtual learning, using the developed electronic education platforms [3].

Along with the merits of e-learning, such as providing greater educational opportunities for students worldwide and improved cost effectiveness [4], some challenges have also been reported in virtual education. For example, the limited numbers of expert instructional designers or instructional technologists to support e-learning processes [5], time zone variations (when conducting real-time distance learning) [6], financial expenses of implementing and maintaining the infrastructure necessary for e-learning [7], lack of face-to-face interaction [8, 9] and time commitment required for teachers to commit to the experience [10] can be mentioned as some of these obstacles. Also, some studies have revealed the non-significant effectiveness of e-learning technologies in comparison with traditional education, in which instruction takes place between an instructor and students who are all physically present in the same space [4, 11].

Among several factors affecting learning yield [12, 13], the spacing effect is one of the most well-established phenomena. Using temporal intervals for re-exposing learners to information over time (spaced learning) leads to more effective retention of knowledge in comparison to presenting it at a single time (massed learning) [14, 15]. It has been proven that presenting the educational contents in a learning process which involves repetition for a second or third time after one or more diverse intervals from the first encounter (spaced learning), has a better outcome as opposed to a state in which the second set of information follows the first immediately in a bolus or mass presentation [16]. As well as the variety of educational methods [17], spacing techniques mainly differ in terms of temporality. Some researchers distribute learning sections over a few days, while others use minutes, hours, weeks or months [18]. Spaced learning has been shown to enhance learning efficiency and retention [19], facilitate skill acquisition and short term and long-term retention in motor skill training [20, 21].

The aim of this study was to increase the effectiveness of virtual education during the COVID-19 pandemic for students. Hence, we designed a virtual spaced learning method to reap the benefits of virtual learning and spaced learning concomitantly. The topic was “Good manufacturing practice (GMP) requirements for pharmaceutical microbiology” in Pharm D students.

## Methods

This study was conducted as an interventional semi-experimental survey among Pharm D students. Pharmacy students in Guilan university of medical sciences in their 9^th^ semester of education who had registered for a pharmaceutical microbiology course were included in this study. Sixty-six students aged 21–24 years old were sorted on a list based on their GPA (grade point average). Next, in order to have similar GPAs in peer groups, odd numbers were categorized as the control group (mass learning) while even numbers formed the study group (spaced learning). This study was performed on the topic of Good Manufacturing Practice (GMP) requirements for pharmaceutical microbiology (microbiological quality control of dosage forms) in a two-hour session. The national integrated virtual education platform (NAVID) was utilized as the matrix for teaching and also exams.

In a face to face briefing session, the teaching method was explained for the students and they were assured that the pre/post-test points would not influence their final point, and consent was obtained. The inclusion criteria for the students were: being a Pharm D student, being in the 9^th^ semester of education (coordinated with normal Pharm D curriculum), registering in a pharmaceutical microbiology course for the first time. The exclusion criteria were: being absent in the briefing session, delay in attending in online session (due to the impossibility of repeating the pre-test), probable network disconnections, lack of interest in attending the study.

**Fig. 1:**
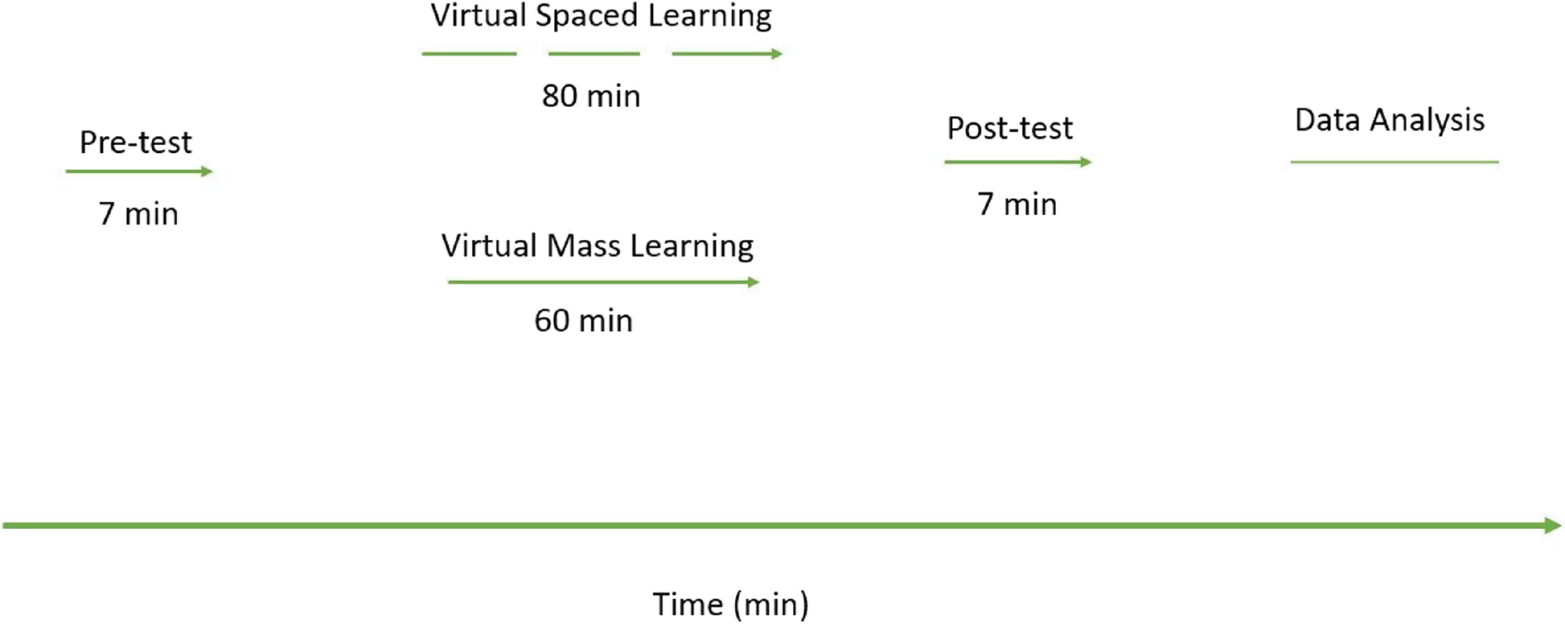
Schematic presentation of implementation of virtual spaced learning method compared to virtual mass learning, along with time requirements

As mentioned, one group was subjected to routine virtual education with narrated power point slides (mass learning) as the control group, while the other group was subjected to the same narrated slides with the difference that two videos were inserted between slides as breaktime (spaced learning study group). Power-point slides were narrated in detail by the instructor for mass virtual education and for spaced learning group, and two diverse 10 min’ videos were placed between slides. The first video was about an overview of a pharmaceutical company and the second one was a meditation video. The two groups of students were registered in NAVID (spaced and mass learning groups) and the classes were conducted on line for both groups as follows: when the students concurrently entered their profile, first the pre-test exam was activated and they had to answer 10 four-choice questions from the scheduled educational content. At the end of the pre-test, the educational content (narrated slides) was activated for the students to watch and listen. After finishing the slides, the post-test exam, which consisted of the same questions as the pre-test was activated, and students had to answer them again. The steps included the following: 1- answering the pre-test, 2- watching and listening to the educational content, 3- answering the post test. The pre/post-test included 10 four-choice questions based on the Kirkpatrick Model [22]. The aforementioned educational process was virtually performed simultaneously for both groups (Fig. 1).

## Statistical analysis

The difference between the average scores of the pre-tests and post-tests of the students in the control and test groups (regardless of the learning method) was determined by the paired t-test (*p* ≤ 0.05) and the difference in educational method efficacy was distinguished by the independent t-test (*p* ≤ 0.05), comparing the groups’ post-test average scores. Data analysis was performed using IBM SPSS Statistics 23 software.

## Results

**Fig. 2:**
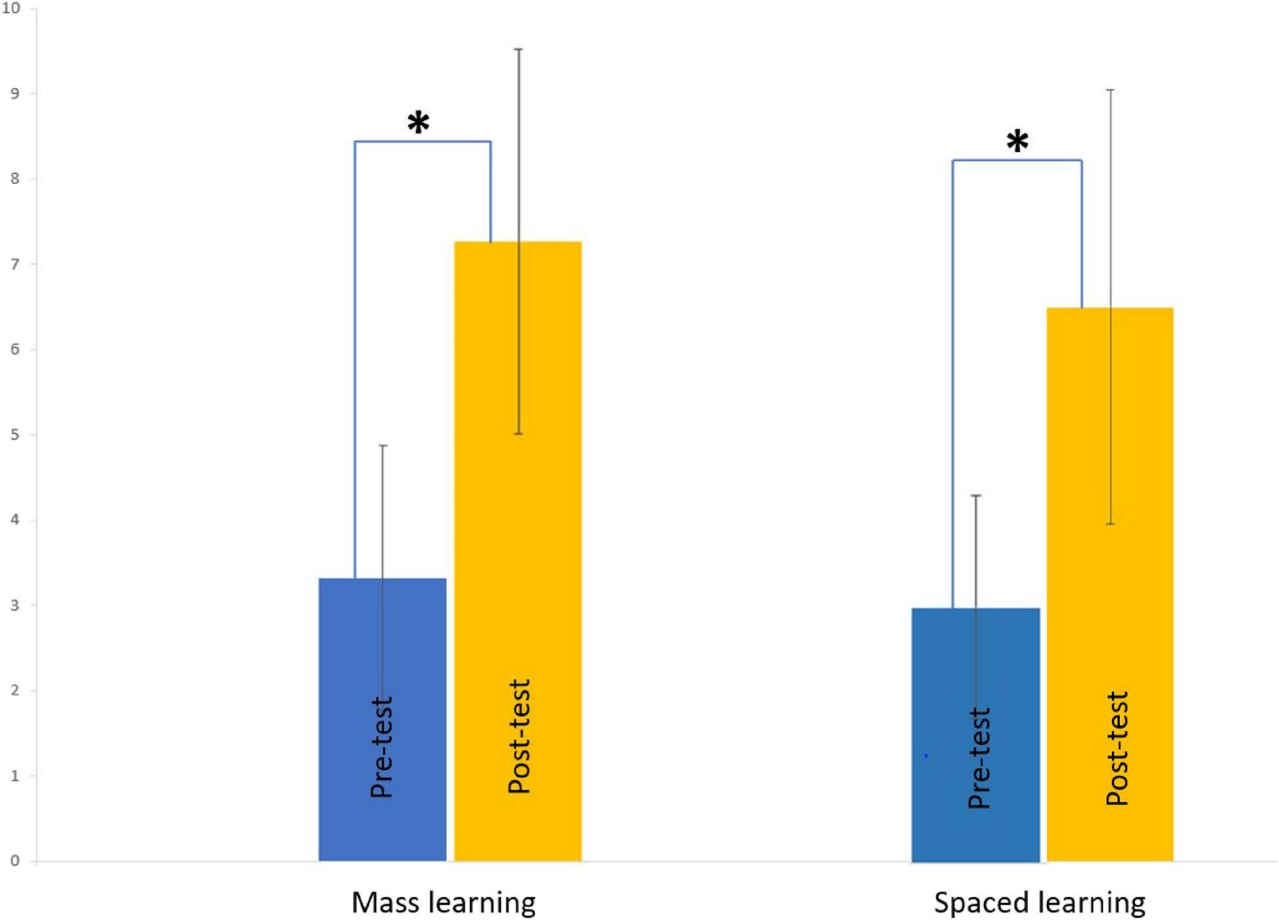
Average points for pre/post-tests in two groups of virtual spaced learning method and virtual mass learning

Amongst 33 existing students in each group, 20 students in the control group (mass learning) and 23 students in the test group (spaced learning) participated in this study. At the end of the teaching process (pre-test, online class, post-test) the average points of the tests were evaluated and compared between the control and test groups. One point was given to each question, so there were 10 points for 10 questions. The minimum and maximum scores of students in the post-test of the mass learning group were 3 and 10, respectively, with an average of 7.26 ± 2.26, and the minimum and maximum scores of students in the post-test of the spaced learning group were 1 and 10, respectively, with an average of 6.5 ± 2.5 as shown in Table 1 and Fig. 2. Results revealed that the average score was not significantly different between the pre-test of the spaced learning (3 ± 1.31) and mass learning (3.32 ± 1.56) methods, and no difference was observed between the two groups’ in the post-test (7.26 ± 2.26 vs 6.5 ± 2.5) utilizing the independent t- test (*p* ≥ 0.05) (Table 2). Variances were considered equal in both groups.

**Table 1:** Pre/post-test average points for spaced learning and mass learning groups

	Pre- test	Post-test
Virtual spaced learning	3 ± 1.31	6.5 ± 2.5
Virtual mass learning	3.32 ± 1.56	7.26 ± 2.26

**Table 2:** Independent t-test between post-tests of spaced learning and mass learning groups

	Levene’s test for equality of variances		T-test for equality of means						
	F	Sig	t	df	Sig. (2-tailed)	Mean dif	Std. error dif	95% confidence interval of the difference	
								Lower	Upper
Post- tests	.721	.401	1.010	39	.319	.763	.756	-.766	2

Based on paired t-test results, the average score was significantly different between the pre-test and post-test (*p* ≤ 0.0001) in both control and test groups, which meant that after the education process utilizing both (mass and spaced) methods, students’ average scores increased in the post-test compared to the pre-test (Table 3).

**Table 3:** Paired t-test for both groups’ pre-test and post-test

	Paired differences					t	df	Sig. (2-tailed)
	Mean	Std. deviation	Std. error mean	95% confidence interval of the difference				
				Lower	Upper			
Mass learning	-3.947	2.592	.595	-5.197	-2.698	-6.638	18	.000
Spaced learning	-3.500	2.721	.580	-4.706	-2.294	-6.033	21	.000

## Discussion

This study was designed and implemented with the aim of comparing the learning effect of two educational methods (mass and spaced education), virtually, on Pharm D students in their 9^th^ semester in a pharmaceutical microbiology course. Results showed that the average points of post-tests were significantly higher than pre-tests in both groups. However, no statistically significant difference was observed regarding the average points of post-tests between the control and test groups.

During the COVID-19 pandemic, online platforms unified the communication and collaboration platforms to allow teachers to create educational courses for training and skill development. Well-known platforms such as Microsoft Teams, Google Classroom, Canvas and Blackboard utilized options like workplace chat, video meeting and file storage that keep classes organized and user friendly [23]. Also, sharing a variety of content, such as Word, PDF and Excel files, audios, videos, and assessment of student learning by using quizzes were possible [24]. However, virtual education was performed through various platforms, and to our knowledge, few studies have utilized them to combine a new teaching technique with e-learning. For example, Doucet et al., have mentioned the flipped classroom as a suitable method for virtual education, whereby learning resources such as articles, pre-recorded videos and YouTube links were provided before the class and the online classroom time was then used to deepen understanding through discussion with faculty and peers [25].

Several studies have been designed to evaluate the mechanism and optimization of spacing and its effect on learning outcomes [26, 27]. Among them, healthcare professionals have widely utilized this technique in theoretical and skill acquiring education [18], including resuscitation courses [28], adaptation of optokinetic response [29], microsurgical procedures [30], emergency medicine [31] and pharmacy [32].

Moreover, in pharmaceutical education, the spaced learning strategy has been incorporated in various areas, including learning the names of drugs [33], performing physical assessments [34], pharmacotherapy [35] and an online spaced-education game for students to study drugs in a skill- lab course [32].

Although many investigations have supported the affirmative effects of spaced learning in pharmacy [36–38], there are some studies which have revealed no significant effect for this learning strategy [39]. Yates et al., presented a case-based, spaced learning strategy for teaching physical assessment skills to first-year pharmacy students as a successful approach [34] and Terenyi et al., confirmed that repeated quizzes with spacing can improve long-term retention of learning in pharmacy students [33]; while Sedlacek et al., observed no improvement in the summative assessment performance of Pharm D students in remote asynchronous lectures including time-spaced quizzes for pharmacotherapeutic courses [35]. In addition, Sando et al., revealed that utilizing an online spaced education game did not significantly affect the scores of pharmacy students in the top 200 drug examination, although, high levels of student engagement and positive student perceptions were observed [32]. These results were in accordance with our findings, which showed no statistically significant difference between students’ scores using the virtual spaced learning method compared with the mass learning method.

This lack of improvement may have been related to the difficulty of the subject taught, which was mainly knowledge-based, or possibly due to the shortness of the course used to assess this teaching method. It seems that clarifying the significant influence of the spaced learning strategy (education and assessment) in pharmacy education, requires further evaluation with careful consideration of the differences between knowledge-based and skill-based courses over a longer time period, and preferentially, in the form of a multi-center or distributed study across several pharmacy colleges coordinately.

## Conclusion

In this study, we aimed to increase the advantages of virtual learning by incorporating the spaced learning method. The virtual spaced learning method offered no statistically significant difference in terms of Pharm D students’ scores compared with the mass learning method in a pharmaceutical microbiology course. A longer period of study is suggested for further evaluation. Also, less complex or skill-based topics may lead to more significant learning among pharmacy students, and this matter also warrants further investigation.

### Supplementary Information

**Additional file 1. **

## Data Availability

All datasets generated for this study are included in this published article [and its supplementary information files].
