# Supplementary material for: Virtual spaced-learning method, during COVID-19 for Pharm D students
Source: BMC Med Educ. 2023 Aug 24;23:605. doi: 10.1186/s12909-023-04595-5 (PMC10463503; doi:10.1186/s12909-023-04595-5)
Supplement: Supplementary file 1 — Additional file 1. [file 12909_2023_4595_MOESM1_ESM.docx]

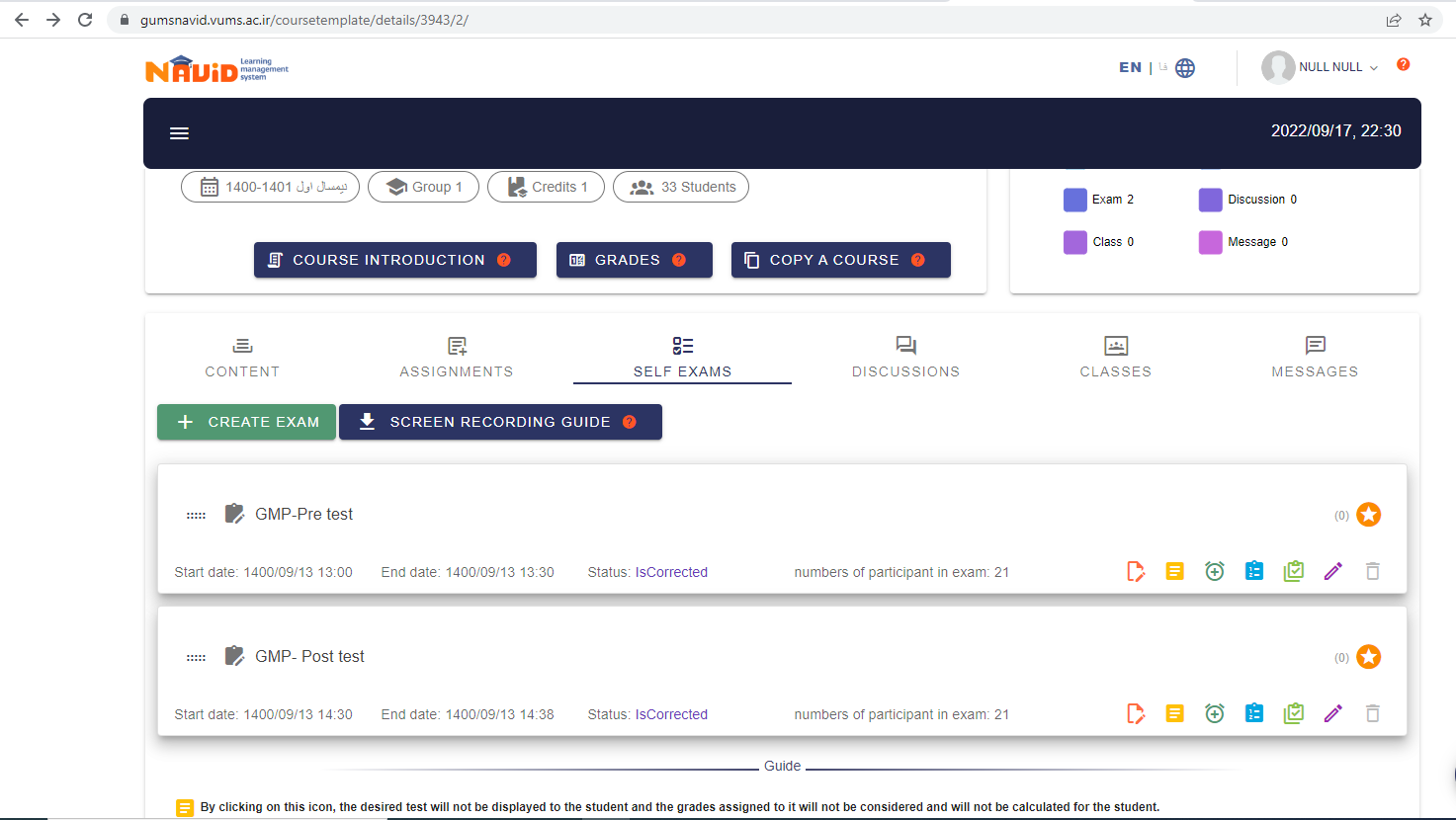


S_1_: Screen shot of online pre/post tests of control group


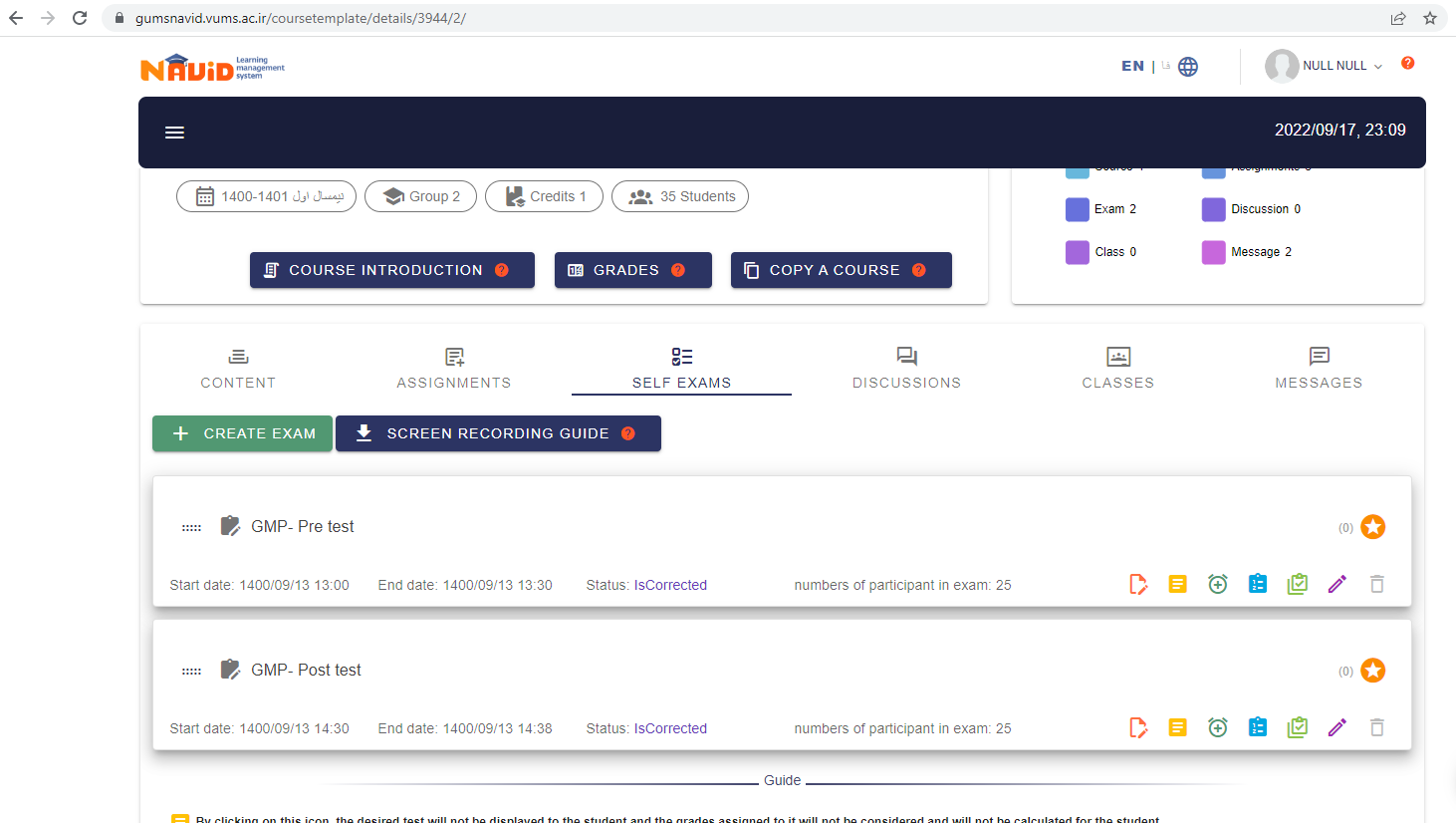


S_2_: Screen shot of online pre/post tests of Spaced learning group
